# Instantaneous radiated power of brain activity: application to prepulse inhibition and facilitation for body dysmorphic disorder

**DOI:** 10.1186/s12938-021-00946-9

**Published:** 2021-10-24

**Authors:** Anastasios E. Giannopoulos, Sotirios T. Spantideas, Christos Capsalis, Panos Papageorgiou, Nikolaos Kapsalis, Konstantinos Kontoangelos, Charalabos Papageorgiou

**Affiliations:** 1 School of Electrical & Computer Engineering, National Technical University of Athens, 9 Iroon Polytechniou Street, Postal Code 15780 Athens, Greece; 2 Department of Electrical and Computer Engineering, University of Patras, Patras, Greece; 3 First Department of Psychiatry, National and Kapodistrian University of Athens Medical School, Eginition Hospital, 74 Vas. Sophias Ave., 11528 Athens, Greece; 4 University Mental Health, Neurosciences and Precision Medicine Research Institute “COSTAS STEFANIS”, (UMHRI), Athens, Greece

**Keywords:** Body dysmorphic disorder, EEG, Instantaneous radiated power, Prepulse inhibition, sLORETA

## Abstract

**Background:**

Global measures of neuronal activity embrace the advantage of a univariate, holistic and unique description of brain activity, reducing the spatial dimensions of electroencephalography (EEG) analysis at the cost of lower precision in localizing effects. In this work, the instantaneous radiated power (IRP) is proposed as a new whole-brain descriptor, reflecting the cortical activity from an exclusively electromagnetic perspective. Considering that the brain consists of multiple elementary dipoles, the whole-brain IRP takes into account the radiational contribution of all cortical sources. Unlike conventional EEG analyses that evaluate a large number of scalp or source locations, IRP reflects a whole-brain, event-related measure and forces the analysis to focus on a single time-series, thus efficiently reducing the EEG spatial dimensions and multiple comparisons.

**Results:**

To apply the developed methodology in real EEG data, two groups (25 controls vs 30 body dysmorphic disorder, BDD, patients) were matched for age and sex and tested in a prepulse inhibition (PPI) and facilitation (PPF) paradigm. Two global brain descriptors were extracted for between-groups and between-conditions comparison purposes, namely the global field power (GFP) and the whole-brain IRP. Results showed that IRP can replicate the expected condition differences (with PPF being greater than PPI responses), exhibiting also reduced levels in BDD compared to control group overall. There were also similar outcomes using GFP and IRP, suggesting consistency between the two measures. Finally, regression analysis showed that the PPI-related IRP (during N100 time-window) is negatively correlated with BDD psychometric scores.

**Conclusions:**

Investigating the brain activity with IRP significantly reduces the data dimensionality, giving insights about global brain synchronization and strength. We conclude that IRP can replicate the existing evidence regarding sensorimotor gating effects, revealing also group electrophysiological alterations. Finally, electrophysiological IRP responses exhibited correlations with BDD psychometrics, potentially useful as supplementary tool in BDD symptomatology.

## Background

The electroencephalography (EEG) measurement has been widely established as a cost-efficient and temporally precise technique for examining the brain activity [1]. Among a wide variety of insights offered by EEG, event-related potential (ERP) technique has been widely used to characterize the normal and pathological neuronal activity [2]. EPRs are typically used to uncover the time-domain cognitive course of information processing by investigating peaks and troughs of the EEG time-series data [3]. Alternatively, the spatio-temporal and spectral features of the EEG are further analyzed via conventional time–frequency decomposition methods aiming at the characterization of the participant’s cognitive profile via EEG markers both in time and frequency domains [4]. To capitalize on the exquisite temporal resolution of EEG, various algorithms have been developed to solve the inverse problem, such as standardized low-resolution electromagnetic tomography (sLORETA) and local autoregressive average (LAURA) [5, 6].

Global measures of brain activity emerge as compression factors towards reducing the dimensions of the analysis and improving the statistical power at the cost of lowering the spatial precision [7, 8]. A global brain measure is commonly derived by averaging or summing across all electrodes, voxels or brain regions, resulting into a univariate representation of single-subject data, usually by a single scalar or time-series. Several whole-brain measures have been proposed in the brain imaging literature for the assessment of normal, pathological and psychiatric neural basis [8–11]. In functional magnetic resonance imaging (fMRI), the global signal (GS) has been used to carry information about widespread neural activity, showing that individual variation in GS topography recapitulates well-established patterns of large-scale functional networks [8]. Using the global brain synchrony (as the spatial coherence of the BOLD signal across regions of the brain) and global metastability (as the extent to which synchrony varies over time), Hellyer et al. [11] showed significant associations between global and localized brain activities.

In the EEG literature, Skrandies [10] has proposed the global field power (GFP) of multichannel EEG recordings as a reference-independent descriptor corresponding to the spatial standard deviation. Complementarily to GFP, the global map dissimilarity (GMD) has been proposed to indicate the topographical change occurring in subsequent potential field distributions [12]. The combined usage of GFP and GMD has proved to be a reliable methodology for the identification of ERP latency and microstate segmentation [13]. Principal component analysis (PCA) has been also used for dimensionality reduction, mainly resulting in three components that account for more than 90% of the variance [14]. Global field synchronization (GFS) was studied in [15] to measure functional synchronization of EEG data in the frequency domain, showing synchronization disconnection for obsessive–compulsive disorder (OCD) patients.

Although the existing EEG global measures can be effectively used for data reduction, there are some key features of their applicability. Notably, GFP only quantifies the extent to which the EEG channels show dissimilar voltage values, assuming that high standard deviation among channels corresponds to increased amount of activity [10]. GMD contains only geometrical information about EEG topographical maps, measuring the geometrical distance between 2 successive EEG maps [12]. PCA can efficiently reduce the dimensions of the EEG data, however it is very sensitive to the pre-processing steps (variance scaling and data standardization) and the selection of principal components [14]. Finally, GFS is a spectral descriptor that only carries information about the degree of global synchronization for a given frequency band [15].

Here, we tested whether the radiational profile of the brain responses could provide an additional whole-brain descriptor. The key motivations to treat the brain as a complex electromagnetic radiator include: (i) the description of the brain responses both in terms of strength (such as GFP and PCA) and synchronization (such as GFS); (ii) the description of the brain activity in the source level, as opposed to the existing scalp-oriented measures and (iii) the representation of brain activity by taking into account the electromagnetic contribution of massive elementary sources. To characterize the radiation signature of the brain, the instantaneous radiated power (IRP) is calculated according to the electromagnetic theory [16–18] and is considered as an overall measure for inspecting the EEG measurements. The IRP calculation is based on the radiational contribution of all current source density (CSD) vectors under the head surface, reflecting the time-course of the brain activity in terms of radiated power. Formally, the IRP computation is a non-linear transformation of the voxels’ activation, proportional to the product of the whole-brain current density and its second derivative. High values of IRP may be associated with the increased in-phase activity of brain sources and resource allocation required for stimulus response.

To test the potency of the proposed methodology, we recorded the EEG from two matched populations (30 body dysmorphic disorder, BDD, patients and 25 control subjects) in a prepulse inhibition (PPI) versus prepulse facilitation (PPF) paradigm [19, 20]. PPI of the startle reflex is defined as the response decrement that occurs when a startling acoustic stimulus (pulse) is preceded immediately by a lower-intensity stimulus (prepulse). On the contrary, PPF refers to the tendency of a subject to increase the startle response when the interval between prepulse and pulse lasts longer than 0.5 s [21]. For these reasons, PPI is considered to reflect early stage of information processing (sensorimotor gating), whereas PPF is associated with later stages of generalized alerting or orienting [20, 21]. Since PPI is thought to enable both global brain activation and synchronization processes, we tested whether the whole-brain IRP could reflect those mechanisms at once. Key motivations to apply the IRP method in this paradigm include: (i) the expected differences in condition, with PPI responses being reduced compared to PPF [22]; (ii) the long-range studies that use the PPI/PFF to contrast the sensorimotor gating effects in psychiatric groups [19]; (iii) the ability of PPI paradigm to be applied in cross-species studies [20], and (iv) the test–retest reliability of PPI/PPF experiments [23].

BDD is a relatively common and often severe psychiatric disorder [24] classified within the spectrum of obsessive–compulsive and related disorders, according to “Diagnostic and Statistical Manual of Mental Disorders” (DSM-5) [25]. This disorder is characterized by distress and excessive preoccupation with one or more perceived defects or flaws in appearance that are not observable or appear only slightly to others. There is a considerable body of evidence suggesting executive dysfunction in BDD and OCD, including deficits in attention, decision-making, sensorimotor gating and cognitive dysregulation [26, 27]. Specifically, BDD patients exhibit different spectral profile (higher theta-1 and reduced beta-1 oscillations), as compared to healthy controls, when they are exposed to PPI/PPF trials [28]. Moreover, N100 and P200 responses evoked by PPI/PPF were investigated in [29] with conventional ERP analysis, showing deficient N100 responses in BDD.

In the present study, we apply the developed IRP methodology on this real EEG dataset, attempting to holistically characterize the neural PPI/PPF responses from a completely electromagnetic perspective. To the best of the authors’ knowledge, purely electromagnetic approaches for whole-brain assessment have not been proposed and examined in the framework of EEG clinical populations. Our hypotheses include that (i) PPI decrement (relative to PPF) may be uniquely reflected in the whole-brain IRP descriptor and (ii) BDD deficits in attentional resource allocation and early-stage processing of the startle reflex may be replicated. For comparison purposes, we conducted two analyses with identical settings, separately using two global brain measures, namely the scalp-oriented GFP and the source-related IRP, as an attempt to investigate their consistency.

## Results

### Simulation results

In this section, we concretely describe the whole-brain measures presented in the Methods using simplified activation waves. To that end, we consider 100 cortical sources oscillating with ideal sinusoidal activations. Without loss of generality, it is assumed that each source oscillates in the *x*-direction with a center frequency of 10 Hz, a random amplitude in the range [0,1] and a random initial phase in the range [0, 2π], depending on the simulation scenario. The simulated activation of the *i*th source may be defined as:

1 $${J}_{x}^{\left(i\right)}\left(t\right)={A}_{i}\cdot \mathrm{sin}\left(2\pi {f}_{c}t+{\varphi }_{i}\right),$$

where ${f}_{c}=10$ Hz is the center frequency, ${A}_{i}$ is the amplitude and ${\varphi }_{i}$ is the initial phase of the sinusoidal. To calculate the IRP, we use the formulas (12, 13) presented in the ‘Methods’ section (constant term ${\mu }_{0}/6\pi c$ is ignored):

2 $$\mathrm{IRP}\left(t\right)=\left(\sum_{i=1}^{100}{J}_{x}^{\left(i\right)}\left(t\right)\right)\cdot \frac{d}{\mathrm{d}{t}^{2}}\left(\sum_{j=1}^{100}{J}_{x}^{\left(j\right)}\left(t\right)\right)=\sum_{i=1}^{100}\sum_{j=1}^{100}{A}_{i}{A}_{j}{\left(2\pi {f}_{c}\right)}^{2}\mathrm{sin}(2\pi {f}_{c}t+{\varphi }_{i})\cdot \mathrm{sin}\left(2\pi {f}_{c}t+{\varphi }_{j}\right).$$

**Fig. 1:**
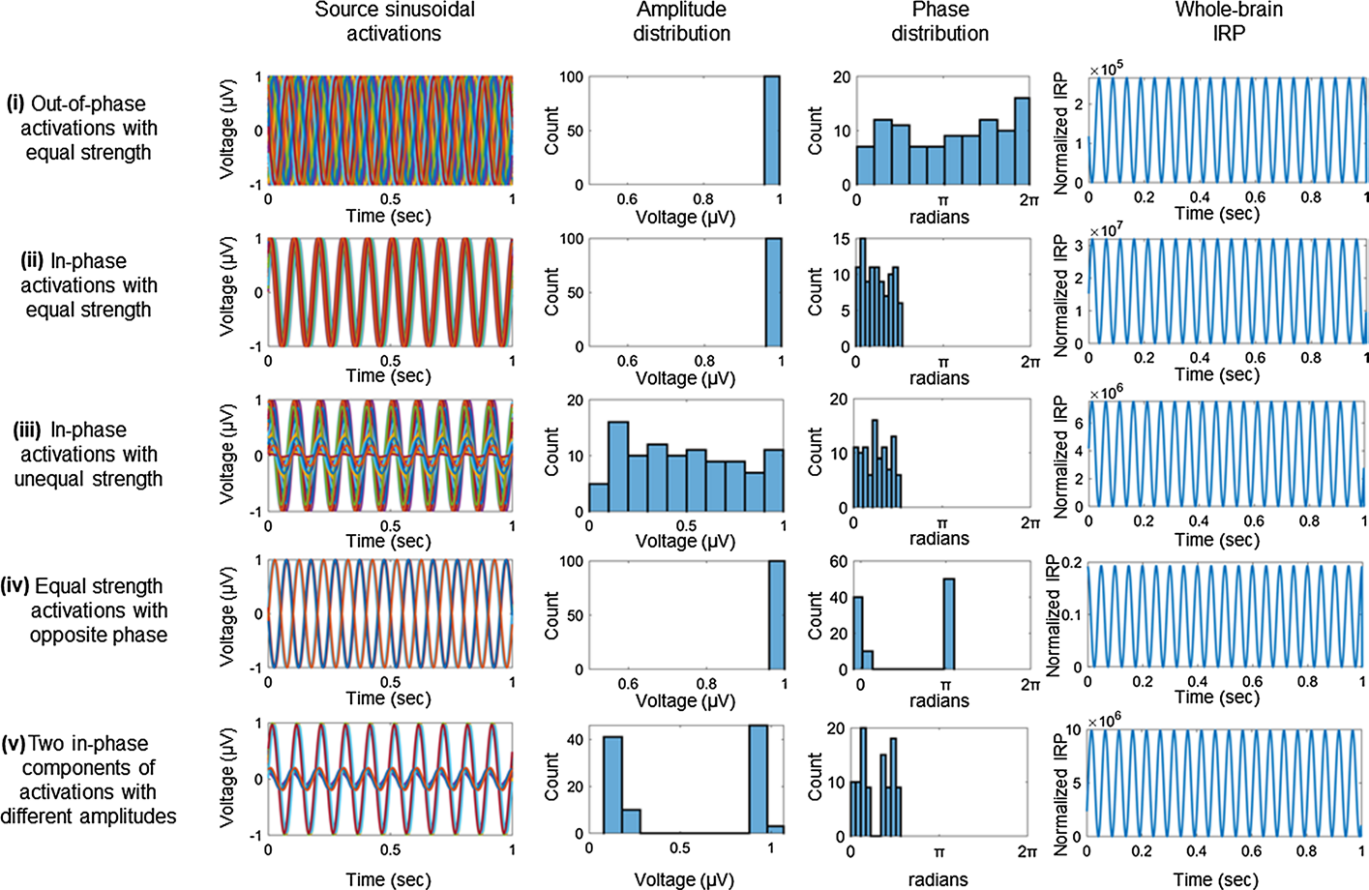
Simulated whole-brain IRP for five different configurations of the source activations. In each scenario (row), the time-course of the source activations, the amplitude and phase distribution of the sinusoidal waves, as well as the IRP time-course are sequentially depicted (columns)

As observed from (2), IRP calculation involves a stretching of the activation by a factor of ${\left(2\pi {f}_{c}\right)}^{2}$, as well as a frequency shift by a factor of 2. Figure 1 demonstrates simulation examples derived from 5 different simulation setups:

i. Whole-brain IRP for out-of-phase activations: all sinusoidal activations have unit amplitude and a random initial phase drawn from the uniform distribution ${\varphi }_{i}\sim U(\mathrm{0,2}\pi )$.
ii. Whole-brain IRP for in-phase activations: all sinusoidal activations have unit amplitude and a random initial phase concentrated in the narrow range of ${\varphi }_{i}\sim U(0,\pi /2)$.
iii. Whole-brain IRP for in-phase activations with varying strength: all sinusoidal activations have different amplitudes drawn from ${A}_{i}\sim U(\mathrm{0,1})$ and a random initial phase concentrated in the narrow range of ${\varphi }_{i}\sim U(0,\pi /2)$.
iv. Whole-brain IRP for opposite activations with equal strength: all sinusoidal activations have unit amplitude and a random initial phase concentrated either in the range of ${\varphi }_{i}\sim U(\mathrm{0,0.1}\pi )$ or ${\varphi }_{i}\sim U(\pi ,1.1\pi )$.
v. Whole-brain IRP for two distinct in-phase components activations: all sinusoidal activations have either near-unit or near-zero amplitude and a random initial phase concentrated in the range of ${\varphi }_{i}\sim U(0,\pi /2)$.

As readily observed from Fig. 1, the whole-brain IRP reaches its maximum amplitude values (in the order of 10^7^) due to the global in-phase activations, oscillating with the maximum amplitude (scenario ii). On the contrary, IRP yields negligible peak values (in the order of 10^–1^) in the case of equal-but-opposite source activations (scenario iv). In scenario i, the IRP is reduced by a factor of 10^2^ in relation to scenario ii due to the phase spread in the source waveforms. Similarly, IRP is also sensitive to the amplitude variations (scenario iii) in the source activations, being reduced by a factor of 10^1^ relative to scenario ii. Finally, scenario v indicates that the IRP is dominated by the high-activation components of source activity, although other components are active and decrease the whole-brain IRP. The simulation analysis indicates a general interdependence between IRP and both phase and amplitude characteristics of the intrinsic dipole activations. Consequently, IRP amplitudes are positively correlated with global brain synchronization and high-strength source activations, while exhibiting restricted spatial information.

### Experiment results

#### Time-windows of ERP components

**Fig. 2:**
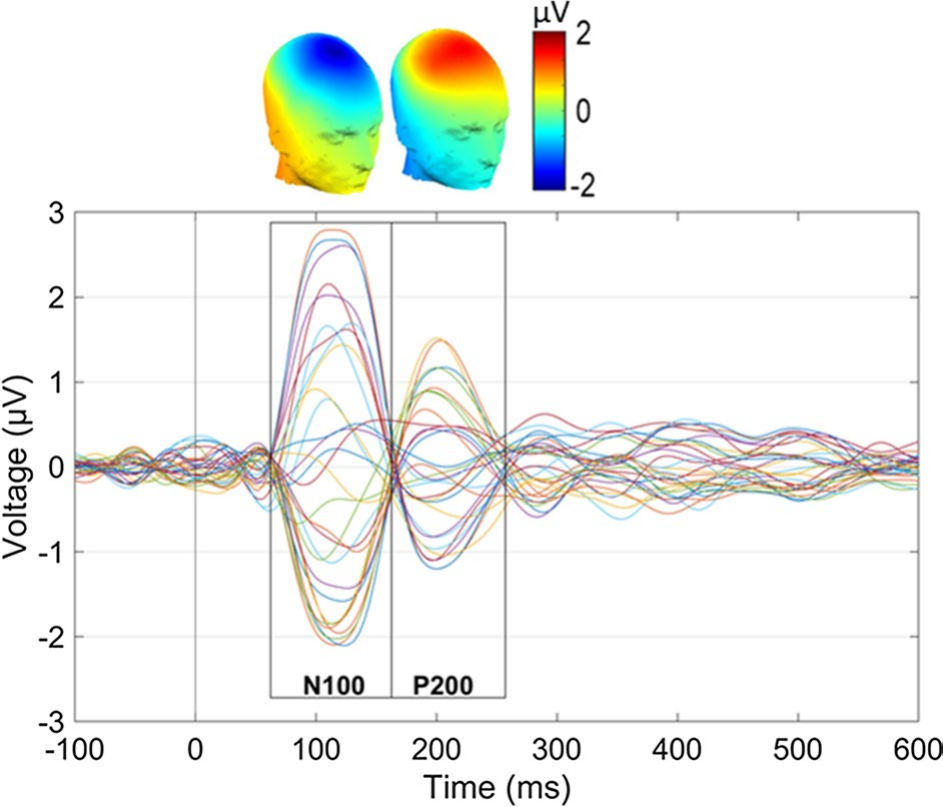
Grand-averaged ERPs across subjects and conditions at all scalp channels. The 3D topographical distribution of N100 and P200 components are also depicted above the respective time-windows. The topographical values correspond to the mean voltage values across 60–160 ms and 161–260 ms time-windows, respectively

Initially, the grand-averaged ERPs (across participants and conditions) were extracted at each channel to visually detect the location and latency of predominant early ERP peaks. This approach, which is called “Collapsed Localizer” in [30], has been used as a preliminary analysis step to visually inspect the dominant ERP components, giving insights about the global task engagement. In line with previous studies on PPI-elicited ERPs (293132), Fig. 2 confirms the presence of two early evoked potentials of the grand-average ERPs, namely the N100 (60–160 ms) and P200 (161–260 ms). Then, the global field power curve (GFP; standard deviation across electrode ERPs at each time point) is computed within the two windows of interest as the baseline global measure. Notably, GFP has been widely used as a reference-independent metric reflecting the global EEG strength in the scalp domain [10, 12].

Both ERP components showed a fronto-central distribution with peak voltage values at the Fz, FCz and Cz channels. In the next sections, we compare the group/condition differences using global descriptors both in the scalp (i.e., GFP) and the source space (i.e., IRP).

#### Global field power

At first, the GFP time-course was extracted separately for each subject and condition [33]. GFP measures were extracted by computing the spatial standard deviation across the scalp channel activity. The calculations were performed separately for the N100 (mean GFP within 60–160 ms) and P200 (mean GFP within 161–260 ms) time-windows, showing the following statistical effects:

*N100 time-window* ANOVA test revealed a significant main effect of condition ($F\left(\mathrm{1,53}\right)=6.70, p=0.012, {\eta }^{2}=0.11$), with PPF (${M}_{\mathrm{PPF}}=2.10, S{E}_{\mathrm{PPF}}=0.09)$ showing higher GFP than PPI (${M}_{\mathrm{PPI}}=1.85, S{E}_{\mathrm{PPI}}=0.09)$. Additionally, there was a significant main effect of group ($F\left(\mathrm{1,53}\right)=6.76, p=0.012, {\eta }^{2}=0.11$), with CTL group (${M}_{\mathrm{CTL}}=2.19, S{E}_{\mathrm{CTL}}=.12$) showing increased GFP than BDD group (${M}_{\mathrm{BDD}}=1.77, S{E}_{\mathrm{BDD}}=0.11$). Finally, no significant interaction effect ($p>0.05$) was observed.

*P200 time-window* ANOVA test showed only a significant main effect of condition ($F\left(\mathrm{1,53}\right)=12.85, p=0.001, {\eta }^{2}=0.195$), with PPF (${M}_{\mathrm{PPF}}=2.04, S{E}_{\mathrm{PPF}}=0.19)$ showing higher GFP than PPI (${M}_{\mathrm{PPF}}=1.63, S{E}_{\mathrm{PPF}}=0.15)$. No other significant effects (${p^{\prime}}s>0.28$) were observed.

**Fig. 3:**
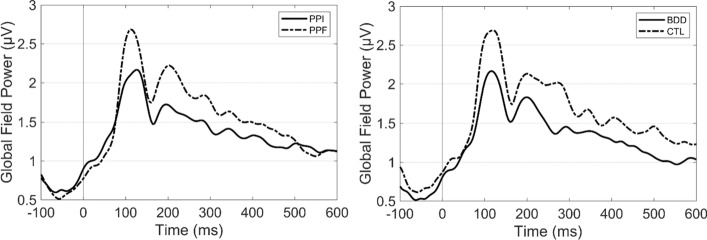
Grand-averaged GFP curves for the main effect of condition (left) and group (right)

Figure 3 depicts the grand-averaged GFP curves, separately computed as the mean across subjects and conditions.

#### Whole-brain IRP

According to the calculation of the proposed IRP measure, each single-subject’s IRP is computed by applying the formulas (12)–(13) (see “Methods” section). This calculation was performed separately for N100 and P200 time-windows.

*N100 time-window* In line with GFP results, the whole-brain IRP during N100 range exhibited significant main effects both in condition ($F\left(\mathrm{1,53}\right)=5.79, p=0.02, {\eta }^{2}=0.10$) and group ($F\left(\mathrm{1,53}\right)=7.75, p=0.007, {\eta }^{2}=0.13$). Specifically, BDD patients ($5.25\times {10}^{9} {\mathrm{A}}^{2}/{\mathrm{mm}}^{2}{\mathrm{ms}}^{2}$) showed reduced IRP compared to CTL group ($11.1\times {10}^{9} {\mathrm{A}}^{2}/{\mathrm{mm}}^{2}{\mathrm{ms}}^{2}$), whereas the PPF responses ($9.19\times {10}^{9} {\mathrm{A}}^{2}/{\mathrm{mm}}^{2}{\mathrm{ms}}^{2}$) were enhanced overall, as compared to PPI ($7.13\times {10}^{9} {\mathrm{A}}^{2}/{\mathrm{mm}}^{2}{\mathrm{ms}}^{2}$). The interaction effect was not significant ($p=.33$).

*P200 time-window* IRP during P200 range yielded a marginal effect on condition ($p=$0.059) and a significant main effect on group ($F\left(\mathrm{1,53}\right)=6.91, p=0.011, {\eta }^{2}=0.12$), whereas the interaction was not significant. BDD ($4.46\times {10}^{9} {\mathrm{A}}^{2}/{\mathrm{mm}}^{2}{\mathrm{ms}}^{2}$) showed again reduced IRP compared to CTL ($7.89\times {10}^{9} {\mathrm{A}}^{2}/{\mathrm{mm}}^{2}{\mathrm{ms}}^{2}$) group, while there was a trend for higher PPF ($6.89\times {10}^{9} {\mathrm{A}}^{2}/{\mathrm{mm}}^{2}{\mathrm{ms}}^{2}$) than PPI ($5.46\times {10}^{9} {\mathrm{A}}^{2}/{\mathrm{mm}}^{2}{\mathrm{ms}}^{2}$) in whole-brain IRP.

**Fig. 4:**
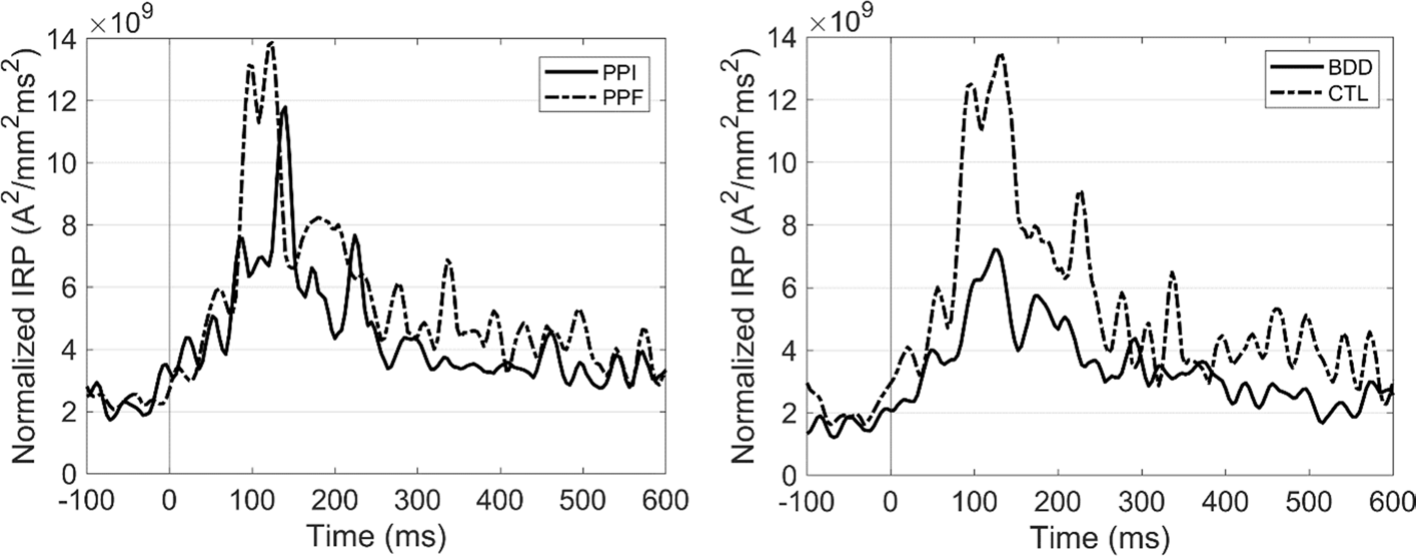
Grand-averaged whole-brain IRP for each condition (left) and group (right)

The grand-averaged IRP waves are illustrated in Fig. 4.

#### Spectral features of IRP

To further investigate the spectral characteristics of the IRP metric, a time–frequency analysis was also conducted. This analysis aimed to identify the predominant frequency components of IRP in order to (i) verify the presence of N100 and P200 components in the frequency domain and (ii) identify the frequency bands that are responsible for the previous time-domain differences. The frequency decomposition of the IRP waveforms was performed via the continuous wavelet transform (CWT) to obtain a reasonable trade-of between temporal and spectral resolution (instead of using FFT which in general has low temporal resolution).

**Fig. 5:**
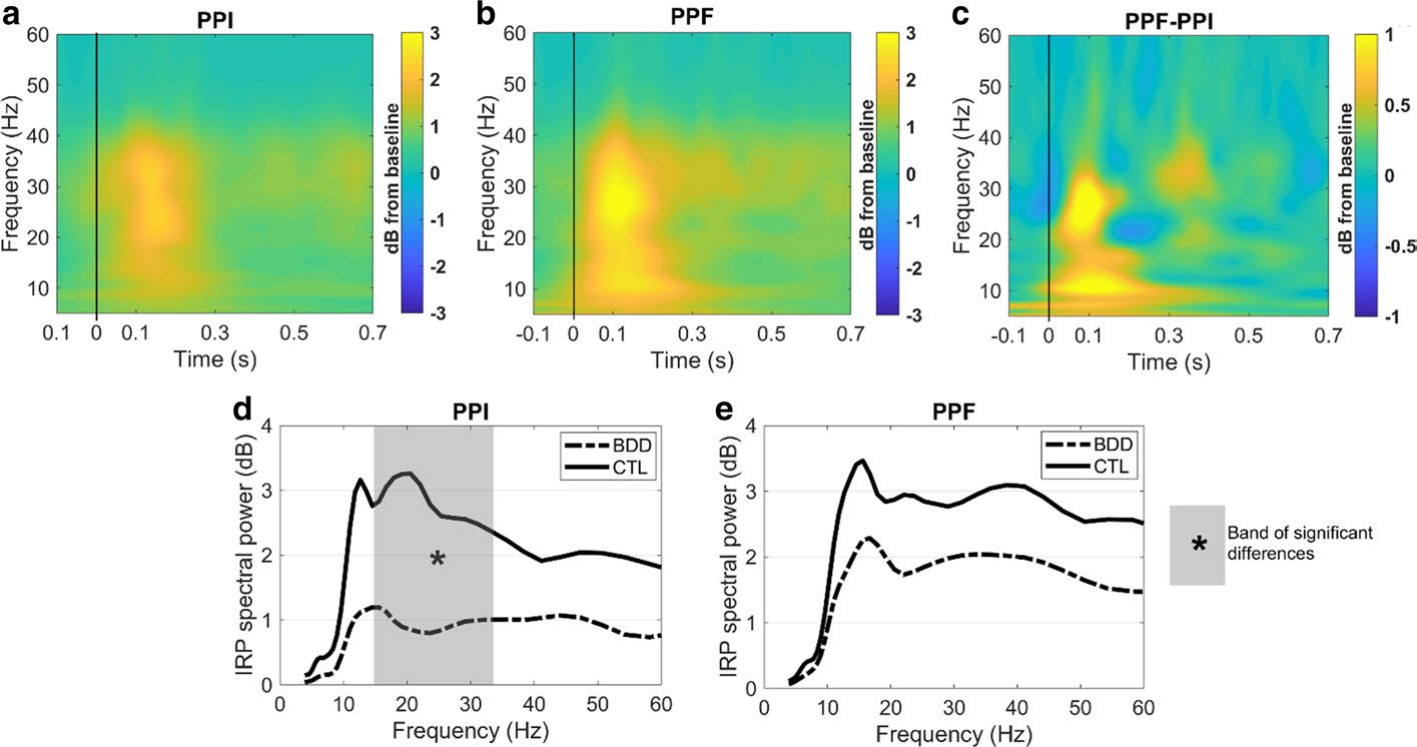
Time–frequency analysis of IRP signals. All IRP spectral values are expressed in dB with respect to the pre-stimulus period (− 0.1 to 0). **A** Grand-averaged time–frequency representations of the IRP in PPI condition. **B** Grand-averaged time–frequency representations of the IRP in PPF condition. **C** Pixel-by-pixel difference between PPF and PPI. From **C**, PPF is greater than PPI responses in two distinct frequency bands (3.8–12.6 Hz and 25.3–31.2 Hz) during the N100/P200 time-windows. **D** Grand-averaged IRP spectral power values in PPI condition for both CTL and BDD groups. The gray-shaded area indicates the frequency band of significant group differences. **E** Grand-averaged IRP spectral power values in PPF condition for both CTL and BDD groups. The IRP spectrum was computed in the 0.6–0.26 s time-window for both **D** and **E**

Figure 5 illustrates the results of the time–frequency analysis. As observed from Fig. 5A, B, the post-stimulus activity of both PPI and PPF is associated with an outburst of the 10–40 Hz frequency band during the N100/P200 time-windows (60–260 ms post-stimulus). Following the cluster permutation testing presented in the Methods (section G.2.), two distinct frequency bands were indicative for the condition differences (see also Fig. 5C for PPF–PPI difference). Specifically, PPF responses were higher than PPI responses in the frequency bands 3.8–12.6 Hz ($p=0.004$) and 25.3–31.2 Hz ($p=0.032$) during the 60–260 ms post-stimulus window. The spectrum waves of CTL and BDD groups were then compared. We found significant group differences only in the PPI condition, with BDD responses being reduced in the band 14.5–33.4 Hz compared to CTL ($p=0.003$). Panels D and E of Fig. 5 show the grand-averaged spectrum waves of both groups in PPI and PPF conditions, respectively. All values are expressed in dB in relation to the pre-stimulus spectral power values.

#### Predictive strength of IRP on psychometrics

Given the remarkable consistency between the effects revealed by the GFP and IRP measures, we further evaluate the correlational relationship between the IRP and BDD psychometrics. Specifically, to test whether the IRP measures could predict either the BDD-YBOCS or DCQ scores, 2 separate stepwise linear regression (SLR) models were conducted. The dependent variables of SLRs were the psychometric scores, while the IRP-N100-PPI, IRP-N100-PPF, IRP-P200-PPI and IRP-P200-PPF responses were considered as predictors.

**Fig. 6:**
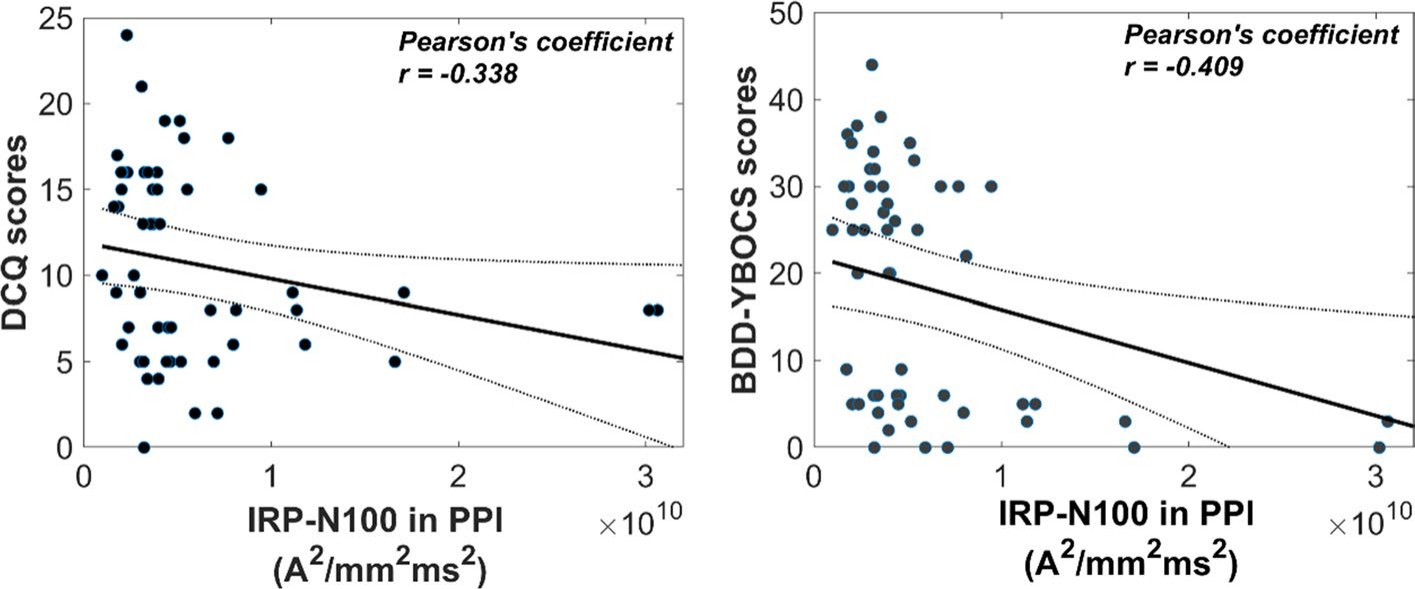
Best-fitting lines resulting from stepwise regression models for DCQ (left) and BDD-YBOCS (right) scores. Gray lines illustrate 95% confidence interval

The models revealed that the IRP-N100 in PPI condition is a significant linear predictor for both DCQ (${R}^{2}=0.114, p=0.0116$) and BDD-YBOCS (${R}^{2}=0.167, p=0.002$). No other terms were predictive on the screening measures. Figure 6 depicts the scatter-graph plots between the IRP-N100 responses in PPI and psychometric ratings.

## Discussion

There are multiple ways to study complex systems, principally by projecting their activity in multidimensional spaces and then searching for local or global descriptors to represent their patterns. This study proposes an electromagnetic methodology to inspect the EEG activity, treating the brain as a complex electromagnetic radiator. After applying source localization of ERP responses, a non-linear transformation of the source data, namely the IRP computation, may be performed to reflect the brain’s radiation profile [16]. To univariately describe the EEG responses, the IRP calculation relies on the radiational behavior of the total current density of the brain taking into account the radiational contribution of all elementary electric dipoles [17, 34].

The developed methodology was used to characterize the electrophysiological activity in response to PPI and PPF trials. To test whether the inhibitory mechanisms and sensorimotor gating effects [22] are globally reflected in the neural activity, two global brain descriptors were evaluated. Specifically, a widely used whole-scalp descriptor (GFP: spatial standard deviation across channels) was firstly examined [10] and the results were contrasted with those derived by following the proposed IRP method.

As a general view, we observe that the inhibitory regulation effect was reflected both in the GFP and IRP responses, with PPI showing significantly reduced amplitudes compared to PPF. PPI and PPF neural responses have been extensively addressed, especially in the electrophysiology literature [22, 29, 31, 32]. Specifically, long-term studies have shown the functionality of PPI mechanism as a diminishment of the startle reflex relative to pulse-alone response, either by evaluating the muscular [35, 36] or neural activity [31, 32]. Interestingly, the condition differences are in line with evidence indicating that PPI and PPF are independent processes [20]. Considering the timing of appeared differences, multiple studies have investigated the electrophysiological responses of PPI in early time-windows, such as the N100 and P200. Indeed, studies [29, 31, 32] have shown reduced distributed LORETA activations during N100/P200 in PPI, attributed to the widespread neuronal networking that supports the inhibitory adjustment. Thus, the presented findings align with the existing evidence, confirming the presence of inhibitory regulation during PPI trials from the global descriptors’ perspective.

In addition, group alterations were observed both using GFP and IRP. Specifically, GFP indexed BDD deficits in the N100 time-window (60–160 ms post-startle), presumably corresponding to impaired performance in attentional orienting [24, 28, 37]. Indeed, given the overlap between OCD and BDD taxonomy [25], have found cortical inhibitory and excitatory dysfunction in patients with OCD [38]. Furthermore, BDD patients exhibit impairments in memory and attention, as demonstrated in several EEG paradigms [29, 37]. Extending the GFP group effect, IRP showed significant differences between control and BDD patients in both N100 and P200 time-windows (60–260 ms post-startle). This finding may constitute an additional electrophysiological marker for BDD, suggesting that the group effects are more elongated in the time-domain representation of the IRP curve.

The consistency in the presented effects between the GFP and IRP is attributed to their similarity in representing a measure that is proportional to the total power of the scalp and source activity, respectively. Specifically, the calculation of GFP is primarily dominated by the sum of squares of channels’ activity, clearly reflecting the scalp-oriented EEG power [10]. Similarly, IRP is based on the summation across voxels representing the total source-domain EEG radiated power. Interestingly, the IRP N100 values in response to PPI trials were found to be significant linear predictor of the BDD severity indicators, namely the BDD-YBOCS and DCQ questionnaires. This finding is also in line with evidence suggesting that BDD severity is negatively correlated with PPI-elicited electrophysiological responses [29, 31].

Noteworthy, EEG activity investigation through the calculation of the IRP time-series is not limited in computations used in this work. Similar to the general EEG analyses, IRP responses may be examined in the frequency and/or time–frequency domain. Such metrics may reflect band-specific brain activation in terms of energetic or power resources required during the mental processing. By extracting a correlation measure between all brain areas of interest that are derived from the IRP curves, cross-frequency or connectivity analyses are also applicable. Goal-driven analyses may take advantage of the IRP metrics in a more precise spatial information, meaning that they may be interested in computing IRP amplitudes within specified brain areas, networks or regions of interest (e.g., DMN, right hemisphere, insula). Other studies may conduct location-wise IRP comparisons by juxtaposing, for example, the IRP emitted by specific brain lobes, regions, Brodmann areas or networks. Since LORETA is usually used as complementary analysis to localize the scalp-oriented effects, IRP computations have to be in line with the first-part analysis. This means that LORETA sources can be extracted either in a trial-by-trial basis or in the averaged-across-trials ERPs, depending on the approach used for the extraction of scalp-oriented measure. Note also that, the generalizability of LORETA-derived sources has to be cautiously interpreted, especially in cases that non-dense (< 32) electrode caps are used for the EEG recordings [see “blurred-localization” effects in [39]. However, since IRP ditches the spatial information of the EEG modality, it is explicitly dedicated for neuroimaging applications requiring high temporal specificity of the measured signals. In this context, IRP may be applied in experiments that mainly attempt to concisely investigate the temporal occurrence of the cognition process, independently of its localized origin. This mainly enables (i) the identification of widespread effects during the cognitive course of information processing and (ii) the determination of time-domain EEG markers discriminating clinical groups. For instance, when considering clinical populations in sensorimotor gating evaluation (such as PPI/PPF paradigms), IRP measure could uniquely reflect global differences among groups, potentially attributed to widespread deficits in sensory systems.

Investigating the whole-brain IRP inherits all the advantages that are already identified in using global brain descriptors; those include dimensionality reduction, holistic representation, reference independence, low-complexity design and statistical robustness. Contradictorily, global measures filter out all the spatial precision of the effects, mainly due to the summation or averaging operation across channels/voxels. In the case of EEG, this drawback is inherently relaxed due to not only the dependency in the reference selection, but also in the restricted precision of source reconstruction algorithms.

## Conclusion

In this study, we proposed an electromagnetic approach tο reflect the radiation profile of brain activity aiming at a global description of the multidimensional EEG data. Simulation analysis showed that the presented IRP time-series is sensitive to the global brain synchronization (in-phase source activation), as well as to the global brain activation strength (IRP amplitude is dominated by high-amplitude sources). To test the potency of the method, the IRP was extracted from two groups (control subjects and BDD patients) in a PPI/PPF paradigm. The GFP curves were also computed for purposes of testing the consistency in the effects revealed by IRP. Overall, we observed that both GFP and IRP replicated the expected differences in conditions, confirming the sensorimotor gating effect, with PPI responses being reduced relative to PPF. Regarding the group differences, we noticed that IRP responses were differentiated between groups in elongated time-windows (both N100 and P200), whereas GFP showed only N100 differences. All group differences revealed reduced responses in the BDD group, potentially linked to the BDD/OCD impairments in attentional resource allocation and dysregulation of gating mechanisms. Importantly, PPI-elicited IRP amplitudes during N100 time-window was negatively correlated with BDD screening measures (DCQ and BDD-YBOCS), potentially helpful as electrophysiological marker to complement BDD symptomatology.

## Methods

### Participants

A total of 55 subjects participated in this study. The BDD group consisted of 30 patients, including 19 females (mean ± SD age of 32.53 ± 8.30 years) and 11 males (mean ± SD age of 27.55 ± 5.77 years). A control group consisting of 25 healthy individuals was matched for age and sex, including 16 women (mean ± SD age of 32.25 ± 9.066 years) and 9 men (mean ± SD age of 27.55 ± 5.65 years). An independent samples t-test confirmed the absence of significant differences between the age of the two groups (*t*(53) = 0.153, *p* = 0.179). Written informed consent was obtained from all participants. Clinical assessment of BDD was performed via clinical interviews by two psychiatrists. BDD was diagnosed according to DSM-5 criteria [25], along with the four supplemental screening measures to confirm the diagnosis: Body Dysmorphic Disorder Examination [40], Yale-Brown Obsessive–Compulsive Scale for BDD [41], Dysmorphic Concern Questionnaire (DCQ) [42] and Brown Assessment of belief scale [43]. Exclusion criteria included active drug or alcohol abuse, history of neurological disorders, and current pregnancy.

### Psychometrics

In order to investigate possible correlations between the electrophysiological (EEG metrics) and behavioral (psychometric scores) data, two different subject-specific scores are available both for BDD and healthy group, mainly assessing the body-related symptoms and proving a total score of BDD severity:

*Yale-Brown Obsessive–Compulsive Scale for BDD (BDD-YBOCS)* This psychometric rating is a specialized, concise and easily administered instrument that measures the severity of BDD symptoms. It is a scale widely used in evaluation of symptom severity and treatment outcome in BDD, recently translated, adapted and validated in Greek [44] resulting in a 12-item rater-administered measure. Each of the 12 items is rated 0–4 points (0 = not at all to 4 = every day) on a Likert scale.

*Dysmorphic Concern Questionnaire (DCQ)* This questionnaire is used for the assessment of dysmorphic concern [42]. Specifically, DCQ is a 7-item self-report measure that assesses cognitive and behavioral symptoms of overconcern with an imagined or slight physical defect. Respondents rate their concern about their physical appearance relative to others on a 4-point scale, ranging from 0 (not at all) to 3 (much more than most people).

BDD group showed 14.17 ± 0.82 (in DCQ) and 29.40 ± 1.04 (in BDD-YBOCS), while controls scored 6.04 ± 0.65 (in DCQ) and 3.80 ± 0.47 (in BDD-YBOCS), with all *t*-tests corresponding to ${p{\prime}}s<0.001$. Notably, the BABS and BDDE measures were obtained only for the BDD group, scoring 18.80 ± 2.80 (in BABS) and 117 ± 27 (in BDDE). Table 1 summarizes the demographics and the psychometrics of both groups.

**Table 1:** Demographics and psychometric ratings of the experimental groups

	BDD group	CTL group	Statistics for BDD-CTL difference	
			t-value	p-value
Number of				
Males	11	9	–	–
Females	19	16	–	–
Total	30	25	–	–
Age (years)				
Males	27.55 ± 5.77	27.55 ± 5.65	t(53) = 0.153	0.439
Females	32.53 ± 8.30	32.25 ± 9.07		
Education (years)	4.73 ± 1.64	5.45 ± 1.73	t(53) = 1.389	0.171
Psychometric scores				
DCQ	14,167 ± 0,815	6040 ± 0,654	t(53) = 7,560	< 0,001
YBOCS-BDD	29,400 ± 1,039	3,800 ± 0,465	t(53) = 21,05	< 0,001
BABS	18.80 ± 2.80	–	–	–
sBDDE	117 ± 27	–	–	–

### EEG acquisition and stimuli

EEG recordings took place in an electromagnetically shielded room. For purposes of minimizing physiological noise, participants sit in a comfortable position and relax before the start of the recording session. The brain signals were amplified (gain 47 dB) by a Braintronics DIFF/ISO-1032 amplifier before entering a 32-bit analogue to digital converter (NI SCB-68). The digitized signal comprised an input for National Instruments PCI-6255 DAQ card (16 bits ADC) through two National Instruments CB-68LP terminal blocks. The PC with the DAQ Card runs a LabView program for the recording of the signals, which can be monitored by an onscreen graphical representation. Evoked biopotential activity was digitalized at a sampling frequency of 1000 Hz from 30 scalp sites (FP1/FP2, FPZ, AFZ, F3/F4, F7/F8, FZ, FC3/FC4, FCZ, FT7/FT8, CZ, T7/T8, CP3/CP4, CPZ, TP7/TP8, P3/P4, P7/P8, PZ, O1/O2, OZ) using active electrodes mounted on an elastic cap according to the International 10–20 System. To enable the detection of blinks and/or saccades, horizontal (HEOG; placed at the outer canthi of the left eye) and vertical (VEOG; placed above the right eye) electro-oculograms were also recorded from two electrodes. Electrode impedance was kept constantly below 5kΩ. Online EEG biopotentials were referenced to the earlobes, while the ground electrode was placed on the left mastoid.

**Fig. 7:**
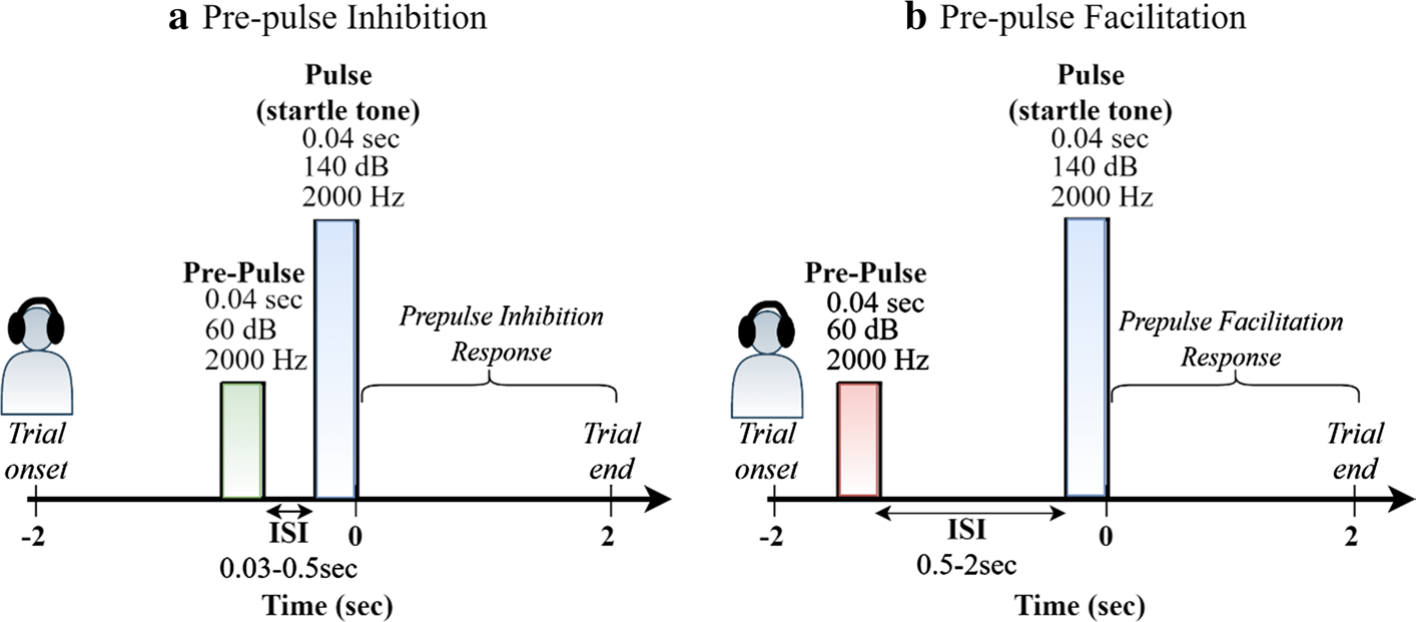
Recording structure of PPI (**A**) and PPF (**B**) trials. The inter-stimulus interval (ISI) between prepulse and pulse varies between 0.03–0.5 s for the PPI trials and 0.5–2 s for the PPF trials. The startle tones and prepulses have an amplitude of 140 dB and 60 dB, respectively. All stimuli have a duration of 0.04 s and a frequency of 2000 Hz

Participants were asked to hear 51 pairs of tones through headphones. The presentation of the trials was random in each session, including 26 prepulse-pulse *short* intervals (PPI, 30–500 ms) and 25 prepulse-pulse *long* intervals (PPF, 500–2000 ms). Each trial recording had a duration of 4 s (− 2 to + 2 s), time-locked to the startle-tone onset. The amplitude of the startling acoustic stimulus (pulse) was 140 dB, while that of the prepulse stimulus was 60 dB. Both stimuli had a frequency of 2000 Hz. Figure 7 depicts the recording structure of PPI and PPF trials.

### Preprocessing pipeline

All datasets were preprocessed using EEGLAB’s (Version 2019.1) denoising functions [45]. Firstly, EEGs were down-sampled to 250 Hz for compression purposes. Offline digital band-pass filtering at 1–40 Hz was performed by using the EEGLAB’s [45] filter function (*pop_eegfiltnew.m*) whose implementation relies on a zero-phase Hamming-windowed sinc FIR filter [46] parameterized as: transition bandwidth = 1 Hz, filter length = 827, roll-off -6 dB/octave. The electrodes showing abnormal time-course were excluded and interpolated. There were no significant differences in the number of rejected channels between groups (*t*(53) = 0.598, *p* = 0.553; 2.80 ± 1.06 channels in BDD; 2.48 ± 0.87 channels in controls). We also visually scanned the single-trial recordings and discarded the trials exceeding $\pm 80 \mu V$ values as excessive blink-related artifacts (BDD group: 2.14 rejected trials per participant; CTL group: 1.93 rejected trials per participant). Subsequently, the scalp activity was re-referenced to the common average.

The resulting datasets were then decomposed via independent component analysis (ICA), providing estimates of component activations. The SASICA tool [47] was used to guide the selection of non-brain (blinks and saccades) components. Component rejection criteria included simultaneous consideration of “Autocorrelation” (weak autocorrelation reflects noisy components), “Focal components” (bad channels have too focal components), “Focal trial activity” (components with focal trial activity correspond to non-brain ones), “EOG correlation” (blink and saccade components are correlated with VEOG/HEOG), “ADJUST” [48] and “FASTER” [49] methods. Before further processing, continuous data were segmented into 4-s epochs (− 2 to + 2 s), time-locked to startle-tone onset, and baseline-corrected based on 0.03 s pre-startle period. This narrow baseline was selected to avoid overlaps with the prepulse tone in the PPI trials.

### Electromagnetics background

In principle, electromagnetic (EM) fields may be distinguished in near and far field zones, depending on the observation distance from a radiating source. In the far field zone, the emitted EM field is described in terms of real power (energy) expressed in watt [17]. Generally, the EM behavior of the brain may be simulated by fitting a large number of elementary dipoles into the cortical matter. Inverse problem algorithms, such as sLORETA, acceptably calculate the equivalent current densities flowing through the dipole sources, each radiating an EM field [5]. These sources may be viewed as a 3D dipole antenna array [17]. The total brain radiation may then be estimated as the superposition of the input power of the dipole array.

Here we present the theoretical background of electromagnetics, according to which the IRP is calculated and extracted from the measured EEGs. Without loss of generality, the current density of each voxel is assumed to be associated to the current of an electric dipole that is only z-orientated (bold notation stands for vector) [18]:

3 $${J}_{z}=I/L,$$

where $L$ is the length (related to the dimensions of the voxel) of the Hertzian dipole fed by current $I$ that is oscillating in the time domain $i\left(t\right)=I\cdot \mathrm{cos}(\omega t)$. By employing spherical coordinates, the theta component of the electric field generated by this elementary oscillating dipole can be expressed [16, 17]:

4 $${E}_{\theta }={j\omega \mu }_{0}\frac{IL}{4\pi }\left[1+\frac{c}{j\omega r}-\frac{{c}^{2}}{{\omega }^{2}{r}^{2}}\right]\frac{{e}^{-j\omega r/c}}{r}\mathrm{sin}\theta ,$$

where $\omega$ is the frequency of the oscillating dipole, $r$ is the observation distance, $\theta$ is the elevation angle, $c$ is the speed of light and ${\mu }_{0}$ is the magnetic permeability of free space. In order to calculate the induced electromotive force on the source, the focus of this analysis is on close proximity distances to the dipole, i.e., $r\to 0$, where the electric field comprises of a singular component and a non-singular component [16]. The latter does not depend on the observation distance r. The exponential term of (4) can be expanded in series:

5 $$\frac{{e}^{-j\omega r/c}}{r}\approx \frac{1}{r} \left(1-\frac{j\omega r}{c}-\frac{{\omega }^{2}{r}^{2}}{2{c}^{2}}+\frac{j{\omega }^{3}{r}^{3}}{6{c}^{3}}\right).$$

In principle, these four terms are sufficient to calculate the non-singular component of the electric field. By substituting (5) in (4), the non-singular component of the electric field may be expressed:

6 $${E}_{{\theta }_{\text{non-singular}}}=-\frac{{(j\omega )}^{2}{\mu }_{0}}{6\pi c}IL\mathrm{sin}\theta $$

or transforming into Cartesian coordinates:

7 $${E}_{{z}_{\text{non-singular}}}=\frac{{(j\omega )}^{2}{\mu }_{0}}{6\pi c}IL.$$

This expression can be generalized in the time domain by employing the inverse Fourier transform:

8 $${E}_{{z}_{\text{non-singular}}}=\frac{{\mu }_{0}}{6\pi c}\frac{{d}^{2}i(t)}{{dt}^{2}}L.$$

By expressing the non-singular electric field parallel to the dipole moment, the electromotive force (induced EMF $\mathcal{E}$) on the elementary dipole may be calculated as the following line integral:

9 $$\mathcal{E}\left(t\right)=-{\int }_{{\varvec{l}}}^{\boldsymbol{ }}{\mathbf{E}}_{\text{non-singular}}\cdot \mathbf{d}\mathbf{l}=\boldsymbol{ }-\frac{{\mu }_{0}}{6\pi c}\frac{{d}^{2}i\left(t\right)}{{\text{dt}}^{2}}{L}^{2}.$$

The instantaneous radiated power of the Hertzian dipole can be then calculated:

10 $${P}_{rad}\left(t\right)=\mathcal{E}\left(t\right)i\left(t\right)=\boldsymbol{ }-\frac{{\mu }_{0}{L}^{2}}{6\pi c}\frac{{d}^{2}i\left(t\right)}{{\text{d}t}^{2}}i\left(t\right).$$

The instantaneous radiated power may be generalized in all spatial orientations taking into consideration the current density vector $\mathbf{J}=\left({J}_{x},{J}_{y},{J}_{z}\right).$ By ignoring the constant term of (10), the 3D instantaneous radiated power may be computed with the following formula:

11 $${P}_{\text{rad}}\left(t\right)=\mathbf{J}\left(t\right)\cdot \frac{{d}^{2}\mathbf{J}\left(t\right)}{{\text{dt}}^{2}}.$$

### Method outline

The proposed method utilizes an inverse-problem (here the sLORETA) algorithm to estimate the CSD vectors that produce the scalp ERP waveforms. The computations of sLORETA rely on a realistic head model based on the MNI-152 template with the three-dimensional solution space restricted to cortical gray matter (5). Before the source localization, ERPs are extracted by separately averaging the condition-specific trials, individually for each single-subject dataset. The 30-channel arrays are then fed into sLORETA-xyz algorithm to calculate the $6239$-voxel source arrays (electric current density of each cube). For each subject, sLORETA outputs the activation of each voxel in the three cartesian dimension (${J}_{X}^{voxel i}(t)$, ${J}_{Y}^{voxel i}(t)$, ${J}_{Z}^{voxel i}\left(t\right), \forall voxel i=1,\dots , 6239$).

**Fig. 8:**
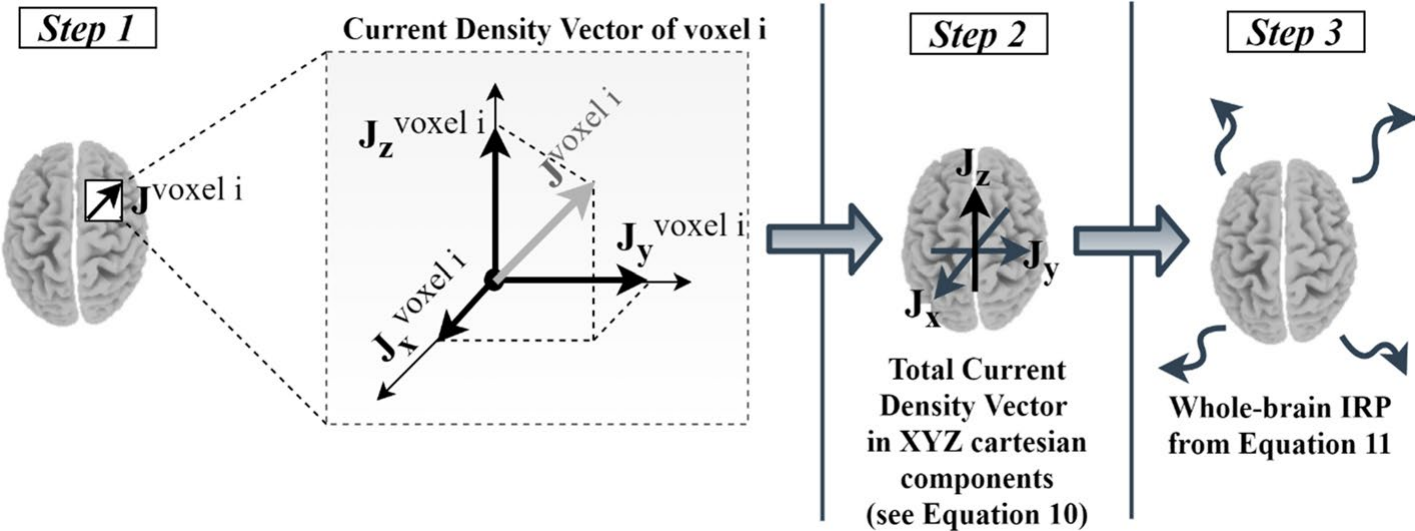
Method outline. In Step 1, sLORETA algorithm returns the time-course of cortical activations as a 3D current density vector. In step 2, the total current density vectors are calculated by summing across voxels, individually for X, Y and Z directions. In step 3, the whole-brain IRP is computed by applying Eq. (13)

The whole-brain IRP computation is outlined following the procedure illustrated in Fig. 8. Specifically, from the sLORETA modeling, each voxel may be considered to contain an elementary electric dipole source, represented by the current density vector and the dimensions of the elementary cube (voxel). According to electromagnetic compatibility theory, the impact of opposite current densities (differential mode currents) of nearly placed voxels to the electric field may be safely neglected, given that the distance between them is very small compared to the dimensions of the problem, such as the wavelength [17, 34]. The significant component of the electric field is generated mainly due to the contribution of the common mode currents (currents of equal sign). Hence, it can be safely assumed that the algebraic summation of the current density vectors over all voxels nullifies the differential mode components [34], while maintaining the common mode components (whole-brain sLORETA activations):

12 $$\begin{array}{c}{J}_{X}\left(t\right)=\sum_{i=1}^{6239}{J}_{X}^{voxel i}\left(t\right),\\ {J}_{Y}(t)=\sum_{i=1}^{6239}{J}_{Y}^{voxel i}\left(t\right),\\ {J}_{Z}\left(t\right)=\sum_{i=1}^{6239}{J}_{Z}^{voxel i}\left(t\right).\end{array}$$

The whole-brain IRP can be then calculated from (11) (dot denotes the time derivative):

13 $${\text{IRP}}\left(t\right)={J}_{X}\left(t\right){\ddot{J}}_{X}\left(t\right)+{J}_{Y}\left(t\right){\ddot{J}}_{Y}\left(t\right)+{J}_{Z}\left(t\right){\ddot{J}}_{Z}\left(t\right).$$

Finally, the total power within the window of interest $[{t}_{1},{t}_{2}]$ may be calculated as the sum of the respective IRP values:

14 $${\text{IRP}}_{[t1,t2]}=\sum_{t={t}_{1}}^{{t}_{2}}{\text{IRP}}\left(t\right).$$

Given that sLORETA algorithm computes the current density $\mathbf{J}=\left({J}_{x},{J}_{y},{J}_{z}\right)$ for each elementary $5\times 5\times 5 {\text{mm}}^{3}$ cube (current density units of $\frac{A}{\text{mm}}$ in each direction), the normalized IRP values are expressed in ${\left(\frac{A}{{\text{mm}}\cdot ms}\right)}^{2}$ and referred to all figures as ‘Normalized IRP’.

### Time–frequency analysis

The time–frequency decomposition of the IRP time-domain signals was performed via the continuous wavelet transform (CWT) in the time period of − 100 to 700 ms (time-locked to the startle-tone onset) [50]. The IRP signals were convolved with complex Morlet wavelets using 78 linearly separated frequencies (from 2 to 80 Hz). The Morlet wavelet kernel used for convolutions with the original IRP signals had a length of 0.5 s, while the kernel was sliding in steps of 4 ms (corresponding to the sampling rate of 250 Hz) until the whole signal is covered. The IRP spectral power at each time–frequency point ($P\left(t,f\right)$) was then computed as the squared absolute value of the respective complex wavelet coefficient [50]. To account for inter-subject variability in spectral power values, the data were dB-normalized (${P}_{\text{dB}}\left(t,f\right)$) based on the pre-stimulus period − 100 to 0 ms (point 0 refers to the pre-prepulse onset) by applying the following formula (for each time–frequency point $(t,f)$):

15 $${P}_{\text{dB}}\left(t,f\right)=10\cdot {\mathit{log}}_{10}\frac{P\left(t,f\right)}{\frac{1}{100}\sum_{i\in \left[-\mathrm{100,0}\right]}P\left(i,f\right)}. $$

Note also that, in a small number of PPF trials (0.93 trials per participant), there was no sufficient 100-ms-long pre-stimulus interval. In those extreme cases, we consider the previous trial’s baseline as the current baseline.

### Statistical analysis

Separately for each of the 2 whole-brain descriptors (GFP and IRP) resulted from the time-domain signals, a two-way mixed analysis of variance (ANOVA) test is conducted to investigate the impact of group (between-subjects factor: BDD vs CTL) and condition (within-subjects factor: PPI vs PPF). Post hoc paired t-tests were conducted to compare the conditions (PPI vs PPF) when a significant main effect of condition was observed. All ANOVA tests refer to Bonferroni and Greenhouse–Geisser corrections to adjust for multiple comparisons and sphericity violations, respectively. Regarding the group comparisons, Levene’s tests were used to confirm the equality of variances between the groups. All statistical procedures were performed using SPSS and MATLAB software. Statistical thresholds were set at *α* = 5%.

*Frequency-domain differences* This section corresponds to the Results section B.4. First, the spectrum waves (from 2 to 80 Hz) during the N100/P200 window (60–260 ms) were derived from the time–frequency representations of the IRP signals. To identify the frequency sub-bands that show significant differences between conditions and groups, we used non-parametric cluster permutation tests [51, 52]. Cluster permutation testing allows to compare the groups/conditions across all frequency points and detect the ‘clusters’ (i.e., consecutive frequency points) of the major differences. It is also appropriate to control for multiple comparisons problem, since the difference distribution is conducted in a data-driven manner using 5000 permutations [52]. In each permutation instance, we randomly shuffle the condition/group labels (‘PPI’ vs. PPF *or* ‘CTL’ vs. ‘BDD’) across the participants. Then, all possible t-tests are performed at each frequency point. The *t*-scores > 2 (corresponding to *p* = 0.05) are then grouped into *clusters* according to whether they belong to successive frequency points. The sum of t-scores within each cluster is then computed to represent the *cluster t-statistic*. Finally, the difference distribution is constructed by using the *maximum cluster t-statistic* of each permutation instance [51]. Original clusters resulted from the actual labels are considered significant if they exceed the critical t-scores (corresponding to *p* = 0.05). The cluster permutation tests were conducted separately for the groups and conditions and allowed us to determine the dominant frequency bands of the N100/P200 differences.

## Data Availability

The datasets and code scripts used and/or analyzed during the presented study are available from the corresponding author on reasonable request.

## Abbreviations

EEG: Electroencephalography
ERP: Event-related potential
sLORETA: Standardized low-resolution electromagnetic tomography
GFP: Global field power
GMD: Global map dissimilarity
PCA: Principal component analysis
GFS: Global field synchronization
OCD: Obsessive–compulsive disorder
IRP: Instantaneous radiated power
CSD: Current source density
BDD: Body dysmorphic disorder
CTL: Control group
PPI: Prepulse inhibition
PPF: Prepulse facilitation
DSM-5: Diagnostic and Statistical Manual of Mental Disorders-Release 5
N100: Negative-going ERP component peaked around 100 ms
P200: Positive-going ERP component peaked around 200 ms
CWT: Continuous wavelet transform
FFT: Fast Fourier transform
SLR: Stepwise linear regression
DCQ: Dysmorphic Concern Questionnaire
YBOCS: Yale-Brown Obsessive–Compulsive Scale
ICA: Independent component analysis
EM: Electromagnetic
EMF: Electromotive force
ANOVA: Analysis of variance
